# Clinical Significance of Antinuclear Antibodies in Patients with Rheumatoid Arthritis: From SETOUCHI-RA Registry

**DOI:** 10.3390/jcm14051553

**Published:** 2025-02-26

**Authors:** Kazuhisa Nakano, Shunichi Fujita, Sumie Hiramatsu-Asano, Akiko Nagasu, Shoko Tsuji, Yuka Koide, Masatomo Yamada, Yo Mizuta, Masakatsu Ikeda, Hiroyasu Hirano, Yoshitaka Morita

**Affiliations:** 1 Department of Rheumatology, Kawasaki Medical School, 577 Matsushima, Kurashiki 701-0192, Okayama, Japan; shunichi@med.kawasaki-m.ac.jp (S.F.); h061eb@med.kawasaki-m.ac.jp (S.H.-A.); nagasu.a@med.kawasaki-m.ac.jp (A.N.); shoko.0513@med.kawasaki-m.ac.jp (S.T.); koidey1223@med.kawasaki-m.ac.jp (Y.K.); yamada.masatomo@med.kawasaki-m.ac.jp (M.Y.); y-mizuta@med.kawasaki-m.ac.jp (Y.M.); mimi2411113@med.kawasaki-m.ac.jp (M.I.); morita@med.kawasaki-m.ac.jp (Y.M.); 2 Department of General Internal Medicine 1, Kawasaki Medical School, 577 Matsushima, Kurashiki 701-0192, Okayama, Japan; h-hirano@med.kawasaki-m.ac.jp

**Keywords:** rheumatoid arthritis, antinuclear antibodies, pulmonary complications

## Abstract

**Background/Objectives:** Rheumatoid arthritis (RA) is a representative systemic autoimmune rheumatic disease (SARD) characterized by synovial inflammation. While antinuclear antibodies (ANAs) positivity in patients with RA varies widely, the relationship between ANA patterns and clinical features remains unclear. This study aimed to evaluate the clinical significance of ANA in patients with RA. **Methods:** This single-center RA registry study included 814 Japanese patients after excluding those with coexisting SARDs. ANA titers and staining patterns were assessed by indirect immunofluorescence assays on HEp-2 cells. Clinical and laboratory features were analyzed, and logistic regression was used to identify risk factors for pulmonary involvement. Hierarchical clustering and statistical analyses were performed to explore associations between ANA patterns and clinical features. **Results:** ANA positivity was observed in 41.5% of patients, with the speckled and homogeneous patterns being the most common. ANA-positive patients exhibited significantly higher rheumatoid factor (RF) and anti-cyclic citrullinated peptide antibody (ACPA) positivity rates and titers, along with elevated disease activity markers, including Evaluator’s Global Assessment and Swollen Joint Count. Nucleolar pattern positivity was independently associated with pulmonary complications, predominantly interstitial lung disease, and higher rates of JAK inhibitor use. Discrete-speckled pattern-positive patients exhibited high ANA titers but lower RF and ACPA levels, reflecting a distinct subset of RA. **Conclusions:** ANA staining patterns and titers are clinically relevant in RA, with nucleolar and discrete-speckled patterns indicating distinct clinical and pathophysiological profiles. ANA should be interpreted alongside other serological markers and clinical parameters rather than as a standalone tool. Further studies are needed to refine its clinical applicability and integration into RA management.

## 1. Introduction

Rheumatoid arthritis (RA) is a representative systemic autoimmune rheumatic disease (SARD) primarily characterized by inflammation of the synovium [1]. If left untreated, RA can lead to joint destruction and irreversible organ damage. Although the etiology remains unknown, autoimmunity with the production of various autoantibodies plays a crucial role in RA-related inflammation. Rheumatoid factor (RF) and anti-cyclic citrullinated peptide antibody (ACPA), both well-recognized autoantibodies in RA, serve as key serological markers used in the diagnosis of RA and as indicators of poor prognosis [1,2].

Antinuclear antibodies (ANA) refer to a diverse group of autoantibodies targeting various nuclear and cytoplasmic components. The indirect immunofluorescence assay (IIFA) using HEp-2 cells has long been recognized as the gold standard for ANA detection. The observed fluorescence patterns reflect different cellular components, including nuclear, cytoplasmic, and mitotic structures. In recent years, the International Consensus on ANA Patterns (ICAP) initiative has established standardized nomenclature and definitions for ANA patterns [3]. ANA fluorescence patterns, along with antibody titers (or fluorescence intensity), are known as significant biomarkers for SARDs, such as systemic lupus erythematosus (SLE) and Sjögren’s syndrome (SS) [4,5]. However, in patients with RA, ANA positivity rates have been reported to vary widely (14–77%) [6,7,8,9]. The relationship between ANA titers or staining patterns (e.g., homogeneous nuclear, speckled nuclear, and cytoplasmic speckled patterns) and clinical manifestations, disease course, or treatment selection in RA remains inconclusive.

To purely assess the clinical significance of ANA in patients with RA, it is essential to exclude patients with coexisting SARDs. The presence of SARDs may influence the frequency of ANA positivity and the clinical manifestations, thereby complicating the interpretation of the specific significance of ANA in RA.

This study aimed to evaluate the differences in clinical characteristics between ANA-positive and ANA-negative patients with RA, specifically excluding those with coexisting SARDs. Additionally, the impacts of ANA titers and staining patterns on disease activity, treatment selection, and prognosis were examined to comprehensively elucidate the clinical significance of ANA in RA.

## 2. Materials and Methods

### 2.1. Patients

All of the study participants were recruited from the prospective comprehensive single-center cohort (SurvEillance of Treatment Outcome of RA patients in Clinics and HospItals: SETOUCHI-RA registry) [10]. All patients are Japanese RA patients, and each has a diagnosis of RA based on the 1987 American College of Rheumatology (ACR) criteria [11] and/or 2010 ACR/European League Against Rheumatism (EULAR) classification criteria for RA [12].

### 2.2. Data Collection

**Figure 1:**
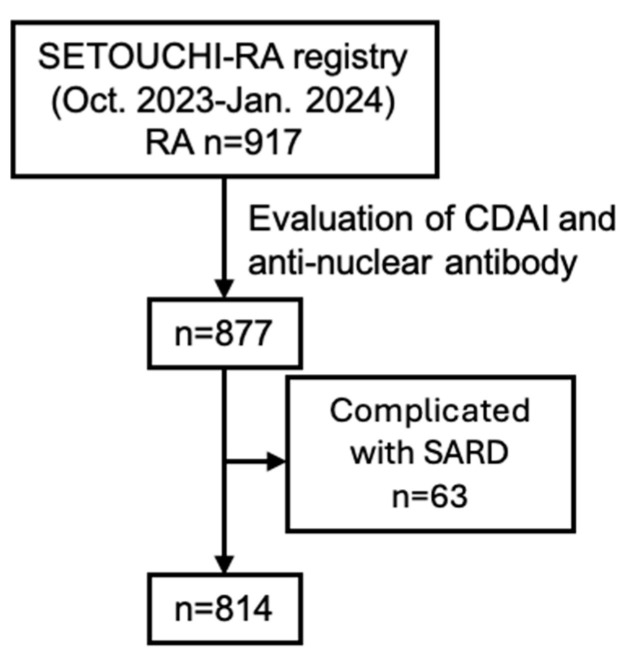
Process of patient selection.

Of the 917 patients with RA aged 18 years or older who visited the hospital between October 2022 and January 2023, 877 patients underwent disease activity assessment using the Clinical Disease Activity Index (CDAI) and serological assessment including ANA. Treatment histories and clinical data were collected with a data-collecting system and evaluated. The 814 patients were included in the analysis, excluding 63 patients with SARDs other than RA (Figure 1, Supplementary Table S1). This study was approved by the Ethics Committee of our hospital (Ethics Application No. 5564-04) and conducted in accordance with the Ethical Guidelines for Medical and Health Research Involving Human Subjects.

Malignancy was defined as the presence of a currently treated or monitored malignant tumor, as well as a history of previously treated malignancy. RA-lung disease was defined as the presence of pulmonary manifestations, including interstitial lung diseases (ILD), airway-related diseases, pulmonary vasculature involvement, and pleural involvement [13,14,15], with the majority of cases being ILD. These conditions were confirmed by a rheumatologist and/or pulmonologist based on clinical evaluation, imaging studies (e.g., chest computed tomography), and/or pulmonary function tests, as appropriate. Difficult-to-treat rheumatoid arthritis (D2T-RA) was defined according to the EULAR criteria [16] as patients who required two or more biologics or JAK inhibitors with different modes of action but still had a CDAI score of 10 or higher, or required glucocorticoids equivalent to 7.5 mg/day or more of prednisolone.

ANA was determined by the fluorescent antibody method (SRL Co., Inc. Tokyo, Japan). Baseline ANA levels more than 1:40 were regarded as positive. ACPA was quantified by the ARCHITECT anti-CCP assay (Abbott Japan, Tokyo, Japan). IgM-rheumatoid factor (RF) was quantified by latex–turbidimetric immunoassay, using the N-Assay LA RF-K Nittobo (Nittobo Medical, Koriyama, Japan). The cutoff levels of the antibodies were set according to the manufacturers’ instructions (ACPA < 4.5 U/mL, RF < 15 IU/mL).

### 2.3. Statistical Analysis

JMP version 17.0 (SAS Institute Japan Ltd., Tokyo, Japan) was used for statistical analyses. Descriptive summary statistics are provided in Table 1 and Supplementary Table S2 for all continuous variables with parametric or non-parametric data, analyzed using the chi-square test or Wilcoxon rank-sum test, as appropriate. Differences were considered statistically significant at a two-tailed *p* < 0.05 level. The clinical information and laboratory data items of patients with RA were subjected to hierarchical clustering using Ward’s method and visualized as a heat map. A multivariable logistic regression analysis was then performed to investigate the factors associated with RA-associated lung disease in the univariable analysis (*p* < 0.1).

RF and ACPA titers were classified into quintiles as follows: RF titer—Q0 (<15), Q1 (15–<33), Q2 (33–<78), Q3 (78–<190), Q4 (≥190); ACPA titer—Q0 (<5), Q1 (5–<34), Q2 (34–<120), Q3 (120–<480), Q4 (≥480).

## 3. Results

### 3.1. Clinical and Laboratory Features in ANA-Positive Patients with Rheumatoid Arthritis

Among the 814 patients with RA analyzed, 338 (41.5%) were ANA-positive, while 476 (58.5%) were ANA-negative. The clinical and laboratory features stratified by ANA positivity are summarized in Table 1. No significant differences were observed in age or disease duration. However, ANA-positive patients were more likely to be female and showed significantly higher positivity rates and titers of RF and ACPA. They also had elevated Evaluator’s Global Assessment (EGA) and Swollen Joint Count (SJ-28). Additionally, ANA-positive patients exhibited higher levels of IgG, IgA, IgM, total protein, and erythrocyte sedimentation rate (ESR), while their albumin/globulin (A/G) ratio was lower. Regarding treatment history, ANA-positive patients were significantly more likely to have used tumor necrosis factor (TNF) inhibitors.

### 3.2. Association Between ANA Staining Patterns and Titers

The most frequent ANA staining patterns were the speckled pattern (313 patients) and the homogeneous pattern (269 patients), as shown in Table 2. Most homogeneous pattern-positive patients also tested positive for the speckled pattern (Supplementary Figure S1). ANA titers for the speckled and homogeneous patterns were predominantly low, with titers of 1:40 observed in 71% and 74% of patients, respectively, and 1:80 in 20% and 19%, respectively. Nucleolar pattern-positive patients showed titers up to 1:80, whereas approximately 60% of discrete-speckled pattern-positive patients exhibited high titers of 1:320 or greater (Table 2).

### 3.3. Relationship Between ANA Titer, Staining Patterns, and Clinical Features

**Figure 2:**
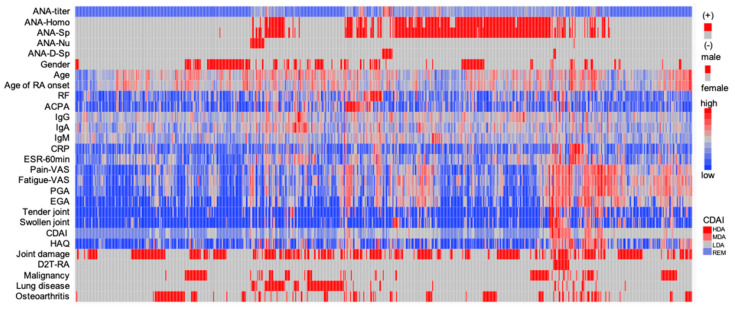
Hierarchical clustering heatmap of clinical and laboratory features in rheumatoid arthritis.

Hierarchical clustering was performed to examine the relationship between ANA titer, staining patterns, and clinical and laboratory features (Figure 2). The heatmap suggests that patient groups with moderate disease activity (MDA) or high disease activity (HDA), including those with D2T-RA, were not consistently associated with ANA positivity. Patients with speckled or homogeneous patterns exhibited a wide range of clinical variables with no clear trends, likely due to the high prevalence of these patterns.

In contrast, nucleolar pattern-positive patients tended to have older age at RA onset, a higher proportion of patients with low disease activity (LDA), and a notable association with pulmonary complications. On the other hand, discrete-speckled pattern-positive patients had higher ANA titers but lower RF and ACPA titers, and their RA disease activity was controlled at CDAI-LDA or below.

When assessing the clinical significance of ANA patterns in ANA-positive patients (Supplementary Table S2), speckled pattern positivity was significantly associated with higher RF positivity and titers, as well as higher ACPA titers. These features were not observed in homogeneous pattern-positive patients. Nucleolar pattern positivity was associated with older age, a higher prevalence of pulmonary complications, and a greater likelihood of JAK inhibitor use. Conversely, discrete speckled pattern positivity was significantly associated with lower RF positivity.

### 3.4. RF and ACPA in Speckled and/or Homogeneous ANA Patterns

**Figure 3:**
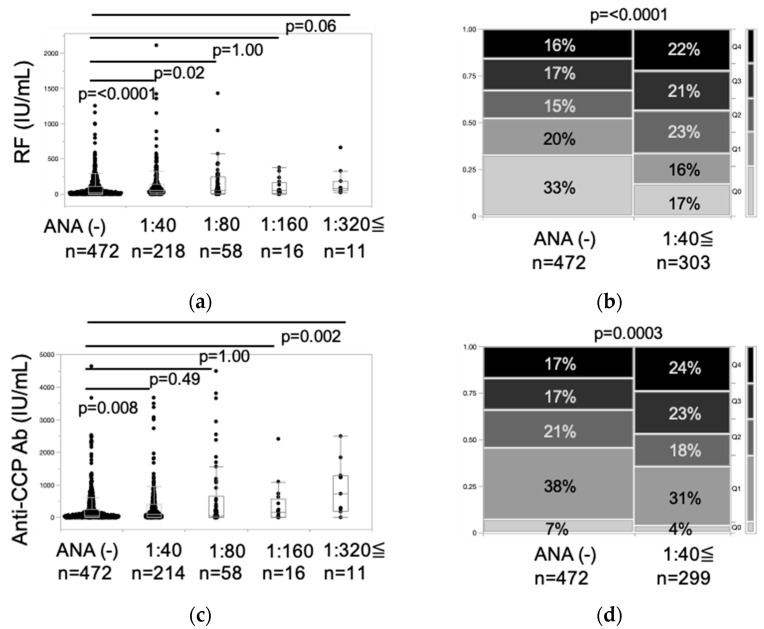
Association between ANA positivity (speckled and/or homogeneous pattern) and RF/ACPA titer in rheumatoid arthritis. For panels (**a**,**c**), non-parametric comparisons with the ANA-negative group were conducted using Dunn’s test. For panels (**b**,**d**), statistical significance was assessed using the exact test for the Cochran–Armitage trend test. In panels B and D, RF and ACPA titers were classified into quintiles as follows: RF titer—Q0 (<15), Q1 (15–<33), Q2 (33–<78), Q3 (78–<190), Q4 (≥190); ACPA titer—Q0 (<5), Q1 (5–<34), Q2 (34–<120), Q3 (120–<480), Q4 (≥480).

Speckled pattern positivity was associated with significantly higher RF positivity and RF and ACPA titers. To further evaluate the relationship, RF and ACPA titers were assessed in ANA-positive patients with speckled and/or homogeneous patterns using both actual values and quartiles (Figure 3). RF and ACPA titers were significantly higher in ANA-positive patients at 1:40 compared to ANA-negative patients (Figure 3a,c). Additionally, in ANA titers of 1:40 or greater, the proportion of RF-negative and ACPA-negative patients decreased by approximately half, while the population in Q3 and Q4 increased (Figure 3b,d).

### 3.5. Risk Factors for Pulmonary Involvement in RA

Pulmonary complications are a critical aspect of RA management. Logistic regression analysis was performed to identify risk factors for pulmonary involvement in RA, including ANA staining patterns. Five variables with *p* < 0.1 in univariate analysis—gender, age, RF titer, ACPA titer, and ANA nucleolar pattern positivity—were included in multivariate analysis (Table 3). The independent risk factors identified were age (odds ratio 1.04, 95% confidence interval (CI) 1.02–1.06) and ANA nucleolar pattern positivity (odds ratio 3.71, 95% CI 1.30–9.82). Among ANA-positive patients, nucleolar pattern positivity was associated with lung involvement, whereas speckled and homogeneous patterns showed no significant correlation with specific extra-articular manifestations.

## 4. Discussion

In this study, we comprehensively evaluated the clinical characteristics of ANA-positive and ANA-negative patients with RA using registry data, and identified several key findings.

First, ANA-positive patients exhibited higher positivity rates and titers of RF and ACPA, suggesting an association with heightened autoimmune activity. Speckled and homogeneous pattern-positive patients demonstrated higher RF and ACPA titers, indicating the potential involvement of these staining patterns in RA pathogenesis. Furthermore, nucleolar pattern positivity was identified as an independent risk factor for pulmonary complications in patients with RA. This finding highlights the potential utility of the nucleolar pattern as a novel marker for assessing pulmonary risk, in addition to conventional RF and ACPA [13,14,15,17,18].

The reported ANA positivity rates in RA range from 14% to 77%, with major differences likely arising from population characteristics (e.g., ethnicity, disease duration) and concomitant treatments, rather than detection methods, as all studies utilized HEp-2 cell-based IIF ANA testing. Although the ICAP has contributed to standardizing ANA interpretation, variability between institutions and among individual observers may still affect results. In our study, the ANA positivity rate was 42%. In contrast, Paknikar et al. reported a 25% ANA positivity rate among patients with RA within 90 days of diagnosis [8], and Liu et al. found a 77% positivity rate in patients with RA compared to other arthritis patients and healthy controls, matched by age and sex [9]. Ethnic and genetic factors significantly influence ANA prevalence and patterns in RA. Studies suggest that ANA positivity is more common in certain populations, such as Asian cohorts, potentially due to genetic predispositions (e.g., HLA associations) [19]. Additionally, ANA positivity is generally more frequent in females and older individuals, reflecting broader autoimmune susceptibility trends. The prevalence of certain autoimmune diseases, including SLE and SS, is also higher in non-white populations, which may contribute to variations in ANA profiles [19]. Additionally, Ishikawa et al. demonstrated that ANA positivity is more likely with TNF inhibitor use [20], emphasizing the need to consider factors such as ethnicity, disease severity, and treatment effects.

TNF inhibitors are known to induce ANA positivity in a subset of RA patients. Previous studies have suggested that this may be mediated through the cross-regulation of TNF-α and interferon (IFN)-α pathways. TNF-α suppresses IFN-α production by inhibiting the differentiation and activation of plasmacytoid dendritic cells [21]. Thus, TNF blockade may lead to sustained IFN-α secretion, which could contribute to ANA induction and, in some cases, lupus-like manifestations [21]. However, it remains unclear whether TNF inhibitor-induced ANA positivity has direct clinical relevance in RA management. Further studies are needed to determine the impact of this mechanism on treatment effectiveness and disease progression.

The interpretation of ANA staining patterns in patients with RA also warrants further discussion. Liu et al. reported that homogeneous patterns were the most common, followed by speckled patterns in 60% of cases [9]. However, in our study, most homogeneous pattern-positive patients were also positive for the speckled pattern. These differences in staining pattern detection may be attributed to variability in interpretation, despite the standardization efforts led by ICAP [3], and could depend on institutional practices or technician expertise [4,5].

Generally, high-titer ANA positivity with homogeneous patterns is strongly associated with SLE, whereas speckled patterns are linked to a variety of autoimmune diseases, including SS and SLE [4,5,22,23]. In our study, patients with RA with other SARDs were excluded, which likely explains the predominance of discrete speckled patterns at high titers (≥1:320). Discrete speckled pattern positivity is almost synonymous with anti-centromere antibody positivity, a disease-specific marker for systemic sclerosis (SSc) [24], and is also a risk factor for primary SS [25]. However, many SSc cases may be limited cutaneous types, making the identification of typical SSc findings challenging [24].

In ANA-positive patients with RA, female predominance, higher RF/ACPA levels, and elevated γ-globulin percentages have been reported [6,9]. In our study, we confirmed these findings, along with higher levels of swollen joint counts and EGA. Although ANA-positive patients with RA without SARD symptoms exhibit normal IL-1β and IL-6 levels compared to healthy individuals, they have significantly higher type I IFN signature gene expression in peripheral blood [26]. This suggests enhanced autoimmune responses mediated by type I IFN signaling [27], which may contribute to the prevalence of homogeneous and speckled patterns in ANA-positive patients. The biological mechanisms underlying ANA positivity in RA are not yet fully elucidated. However, recent studies suggest that type I IFN signaling plays a critical role in the autoimmune process, contributing to both ANA production and disease pathogenesis, particularly in genetically susceptible individuals [27]. Future research should focus on clarifying the interplay between ANA and RA-specific immune pathways to establish its role in disease progression and treatment response.

For the evaluation of connective-tissue disease-associated ILD, markers such as RF, ACPA, anti-Scl-70 and anti-melanoma differentiation-associated gene 5 (MDA5), anti-tRNA synthetase antibodies are considered useful [28]. Nucleolar pattern positivity is strongly associated with SSc and related autoantibodies, such as anti-U3 RNP, anti-Th/To, anti-NOR 90, and anti-PM/Scl antibodies [29]. Furthermore, while nucleolar ANA positivity was associated with pulmonary complications, we found no strong correlation between other ANA patterns and extra-articular organ involvement. This highlights the need for further studies to delineate the clinical implications of different ANA staining patterns in RA. Among patients with RA with ILD, nucleolar pattern positivity may suggest the presence of an SSc spectrum disease, which could influence therapeutic decisions. Recent studies have demonstrated the utility of JAK inhibitors in RA with ILD [30,31,32]. Patients with nucleolar pattern positivity had a significantly higher rate of JAK inhibitor use, possibly reflecting a higher prevalence of pulmonary complications in this subgroup. In contrast, ANA-positive patients with speckled or homogeneous patterns were more likely to receive TNF inhibitors, possibly due to their higher RF and ACPA titers. However, our study does not assess treatment effectiveness, and further prospective studies are needed to confirm these associations. Nonetheless, whether JAK inhibitors are optimal in the context of SSc spectrum diseases warrants further investigation.

Discrete speckled pattern-positive patients exhibited high ANA titers but low RF/ACPA levels, suggesting a distinct pathophysiological subset among patients with RA. Clinical differences between ANA-positive patients with speckled or homogeneous patterns and those with discrete speckled or nucleolar patterns underscore the potential utility of ANA staining patterns in clinical practice and therapeutic decision-making.

This study has several limitations. First, ANA detection was performed using the IIF method, which is subject to variability in staining pattern interpretation due to institutional practices or technician experience. Future studies should incorporate specific ANA subtypes, such as anti-SSA, anti-dsDNA, and antiphospholipid antibodies, using highly specific assays such as immunoblot, ELISA, or Crithidia luciliae indirect immunofluorescence test (CLIFT). This would provide a more detailed immunological profile and further clarify the clinical implications of ANA positivity in RA patients. As a single-center study, generalizability may be limited, and multi-center studies are needed to validate our findings. Furthermore, due to the cross-sectional design, we could not assess the longitudinal impact of ANA positivity on disease progression and treatment response. Additionally, the effects of treatment on ANA results cannot be fully excluded, especially considering reports that TNF inhibitors can induce ANA positivity. Future prospective studies should address these gaps to refine the clinical significance of ANA in RA. Furthermore, while this study excluded patients with multiple coexisting SARDs, secondary SS is relatively common in RA and can be challenging to diagnose accurately [33]. We focused on the primary disease states of SARD and did not treat secondary SS as an independent condition. In our RA registry, if other SARDs were diagnosed based on their respective classification or diagnostic criteria, they were counted as SARDs coexisting with RA. However, we did not comprehensively evaluate detailed autoimmune features. Additionally, when diagnosing RA based on the 2010 ACR/EULAR classification criteria [12], the presence of a confirmed SARD diagnosis can make it unclear whether the case truly represents RA. To ensure that we analyzed a population that could be diagnosed as “pure RA”, we excluded patients with other diagnosed SARDs from this study. Understanding the changes in ANA and other immune profiles caused by secondary autoimmune conditions remains an important area for future research.

To clarify the clinical significance of ANA in RA, multicenter prospective studies with larger cohorts, including treatment-naïve patients, are necessary. As this study is cross-sectional, it cannot determine whether ANA positivity actively contributes to disease progression or is simply a marker of immune activation. Longitudinal studies are required to evaluate its predictive value in disease progression and treatment response. Future research should also explore the biological mechanisms linking ANA positivity to disease activity and treatment response.

## 5. Conclusions

In this study, we demonstrated that ANA-positive RA patients exhibit distinct clinical and serological characteristics, with specific ANA staining patterns correlating with disease activity and pulmonary complications. While our findings suggest that ANA positivity may serve as a potential biomarker for patient stratification, its clinical applicability remains uncertain. Further studies incorporating specific autoantibody subtypes and longitudinal analyses are needed to refine its role in RA management. ANA testing should be interpreted in the context of other serological markers and clinical features, rather than as a standalone diagnostic or prognostic tool. Future research should explore the prognostic implications of ANA in RA and assess its role in guiding therapeutic decision-making, with the goal of integrating ANA testing into personalized RA management strategies.

## Figures and Tables

**Table 1:** Clinical and laboratory features in patients with rheumatoid arthritis stratified by anti-nuclear antibody status.

	Total	ANA-Negative	ANA-Positive	*p*-Value
Number	814	476 (41.5)	338 (58.5)	
Gender: Female (%)	613 (75.3)	346 (72.7)	267 (80.0)	0.048
Age, y	66 (56, 75)	66 (57, 74)	66 (55.8, 75)	0.83
Disease duration, M	131 (67, 200)	129 (69, 191)	137 (61, 203)	0.39
BMI, kg/m^2^	22.5 (20.0, 25.0)	22.6 (19.9, 24.8)	22.5 (20.1, 25.4)	0.55
RA Family history (%)	164 (20.3)	92 (19.4)	72 (21.5)	0.48
Smoking history (%)	319 (39.2)	192 (40.5)	127 (37.6)	0.42
Positivity of RF (%)	594 (73.0)	321 (67.4)	273 (80.8)	<0.0001
Positivity of ACPA (%)	629 (77.3)	352 (74.0)	277 (82.0)	0.007
Erosive X-ray (%)	416 (51.4)	238 (50.2)	178 (53.0)	0.31
Pain VAS	15 (2, 40)	10 (2, 30)	20 (2, 40)	0.21
Fatigue VAS	20 (10, 50)	20 (10, 50)	27.5 (10, 50)	0.41
PGA	20 (5, 40)	17 (3, 30)	20 (10, 40)	0.12
EGA	0 (0, 10)	0 (0, 10)	10 (0, 10)	0.04
TJ	0 (0, 0)	0 (0, 0)	0 (0, 0)	0.91
TJ28	0 (0, 0)	0 (0, 0)	0 (0, 0)	0.84
SJ	0 (0, 0)	0 (0, 0)	0 (0, 0.25)	0.09
SJ28	0 (0, 0)	0 (0, 0)	0 (0, 0)	0.03
CDAI	3 (1, 6)	2 (1, 6)	3 (1, 7)	0.17
HAQ	0 (0, 0.5)	0 (0, 0.5)	0 (0, 0.625)	0.81
EQ-5D	0.77 (0.65, 1)	0.77 (0.67, 1)	0.77 (0.65, 1)	0.68
D2T-RA (%)	24 (2.9)	14 (2.9)	10 (3.0)	1.00
Complication				
Lung disease (%)	106 (13.0)	65 (13.7)	41 (12.1)	0.60
Malignancy (%)	137 (16.8)	80 (16.8)	57 (16.9)	1.00
Osteoarthritis (%)	180 (22.1)	107 (22.5)	73 (21.6)	0.80
Mental disorders (%)	34 (4.2)	17 (3.6)	17 (5.0)	0.37
Laboratory data				
RF, IU/mL	42 (10, 135)	29.5 (0, 121)	54 (21, 163)	<0.0001
ACPA, U/mL	60.9 (6.7, 339)	47.5 (4.0, 255)	81.1 (10.8, 429.4)	0.0009
ESR, mm/h	14 (6, 29)	12 (5, 25)	18 (8, 33)	<0.0001
CRP, mg/dL	0.12 (0.05, 0.33)	0.11 (0.04, 0.32)	0.12 (0.06, 0.33)	0.33
WBC, /μL	5510 (4435, 6875)	5480 (4460, 6863)	5640 (4390, 6900)	0.98
Hb, g/dL	13.1 (12.2, 14)	13.1 (12.2, 14)	13.1 (12.1, 14)	0.28
Plt, ×10^3^/μL	232 (190, 278)	232 (190.8, 208.3)	230 (190, 273)	0.96
TP, g/dL	7.1 (6.9, 7.4)	7.1 (6.8, 7.4)	7.2 (6.9, 7.5)	<0.0001
Alb/Glob ratio	1.4 (1.2, 1.6)	1.4 (1.2, 1.6)	1.3 (1.2, 1.5)	<0.0001
IgG, mg/dL	1257 (1069, 1473)	1196 (1032, 1406)	1353 (1136, 1598)	<0.0001
IgA, mg/dL	256 (186, 341)	247 (174, 331)	266 (205, 354)	0.003
IgM, mg/dL	89 (64, 126)	82 (57, 113)	102 (75, 141)	<0.0001
Previously Used DMARDs				
MTX (%)	717 (88.1)	423 (88.9)	294 (87.0)	0.44
bDMARDs (%)	478 (58.7)	267 (56.1)	211 (62.4)	0.07
TNFi (%)	391 (48.0)	212 (44.5)	179 (53.0)	0.02
IL-6Ri (%)	167 (20.5)	108 (22.7)	59 (17.5)	0.08
ABT (%)	94 (11.5)	49 (10.3)	45 (13.4)	0.22
JAKi (%)	125 (15.4)	74 (15.6)	51 (15.1)	0.92
Glucocorticoid (%)	524 (64.4)	312 (65.6)	212 (62.7)	0.41
DMARDs in Current Use				
MTX (%)	498 (61.2)	295 (62.0)	203 (60.1)	0.61
TNFi (%)	169 (20.7)	83 (17.4)	86 (25.4)	0.01
IL-6Ri (%)	96 (11.8)	65 (13.7)	31 (9.2)	0.06
ABT (%)	56 (6.9)	31 (6.5)	25 (7.4)	0.67
JAKi (%)	103 (12.7)	60 (12.6)	43 (12.7)	1.00
Glucocorticoid (%)	148 (18.2)	93 (19.5)	55 (16.3)	0.27

Data are median (IQR, Interquartile range) except where noted. BMI = body mass index; RA = rheumatoid arthritis; RF = rheumatoid factor; ACPA = anti-citrullinated peptide antibody; VAS = visual analog scale; PGA = physician’s global assessment; EGA = evaluator’s global assessment; TJ = tender joint; SJ = Swollen joint; CDAI = clinical disease activity index; HAQ = Health Assessment Questionnaire; ESR = erythrocyte sedimentation rate; CRP = C-reactive protein; DAS = disease activity score; DMARDs = disease-modifying anti-rheumatic drugs; MTX = methotrexate; bDMARDs = biologic DMARDs; TNFi = TNF inhibitors; IL-6i = IL-6 inhibitor; ABT = abatacept; JAKi = JAK inhibitor.

**Table 2:** The pattern and titer of immunofluorescence staining for antinuclear antibodies in patients with rheumatoid arthritis.

	ANA Positive Number	ANA-Titer, Number (%)					
		1:40	1:80	1:160	1:320	1:640	1:1280
Homogeneous	269	199 (74)	51 (19)	14 (5)	5 (2)	0 (0)	0 (0)
Speckled	313	223 (71)	63 (20)	16 (5)	9 (3)	2 (1)	0 (0)
Nucleolar	19	11 (58)	8 (42)	0 (0)	0 (0)	0 (0)	0 (0)
Discrete Speckled	16	1 (6)	2 (13)	2 (13)	6 (38)	3 (19)	2 (13)

ANA = anti-nuclear antibody.

**Table 3:** Clinical risk factors for lung involvement in rheumatoid arthritis.

	Univariable				Multivariable			
	Odds Ratio	95% CI		*p*-Value	Odds Ratio	95% CI		*p*-Value
		Lower	Upper			Lower	Upper	
Gender; male	1.61	1.027	2.485	0.038	1.32	0.822	2.094	0.245
Age	1.04	1.024	1.061	<0.0001	1.04	1.018	1.057	<0.0001
BMI	0.96	0.912	1.016	0.171				
RA family history	0.68	0.377	1.171	0.172				
Smoking history	1.27	0.839	1.917	0.256				
Drug allergy	1.17	0.668	1.963	0.569				
RF titer	1.00	1.001	1.002	0.001	1.00	1.000	1.002	0.056
ACPA titer	1.00	1.000	1.001	0.003	1.00	1.000	1.001	0.059
ANA-Speckled	0.84	0.543	1.279	0.419				
ANA-Homogeneous	0.77	0.487	1.203	0.260				
ANA-Nucleolar	4.10	1.495	10.447	0.008	3.71	1.303	9.817	0.016
ANA-D-Sp	0.44	0.024	2.206	0.373				

CI = confidence interval; BMI = body mass index; RA = rheumatoid arthritis; RF = rheumatoid factor; ACPA = anti-citrullinated peptide antibody; ANA = anti-nuclear antibody; D-Sp = discrete speckled.

## Data Availability

All data generated or analyzed during the study are included in this published article.

## Supplementary Materials

The following supporting information can be downloaded at: https://www.mdpi.com/article/10.3390/jcm14051553/s1, Table S1: Complicated systemic autoimmune arthritic diseases; Figure S1: Venn diagram of positive speckled and homogeneous patterns; Table S2: Clinical and laboratory features given by anti-nuclear antibody staining patterns in ANA-positive patients with rheumatoid arthritis.
