# Supplementary material for: Clinical Significance of Antinuclear Antibodies in Patients with Rheumatoid Arthritis: From SETOUCHI-RA Registry
Source: J Clin Med. 2025 Feb 26;14(5):1553. doi: 10.3390/jcm14051553 (PMC11900084; doi:10.3390/jcm14051553)
Supplement: Supplementary file 1 [file jcm-14-01553-s001.zip › jcm-3477573-supplementary.pdf]

Table S1

Complicated systemic autoimmune arthritic diseases

	ANA negative	ANA positive
Total	18	45
SLE/DLE	1 / 0	3 / 0
Sjogren syndrome	3	23
Myositis	6	5
Systemic sclerosis	0	9
PMR/RS3PE	1 / 1	2 / 1
Psoriatic arthritis	0	1
SAPHO	1	0
Behçet's disease	0	1
Vasculitis	3	1
others	2	0

There are some overlaps.
ANA: anti-nuclear antibody, SLE: Systemic Lupus Erythematosus, DLE: Discoid Lupus Erythematosus, SAPHO: Synovitis, Acne, Pustulosis, Hyperostosis, and Osteitis, PMR: Polymyalgia Rheumatica, RS3PE: Remitting Seronegative Symmetrical Synovitis with Pitting Edema

Figure S1

Venn Diagram of Positive Speckled and Homogeneous Patterns

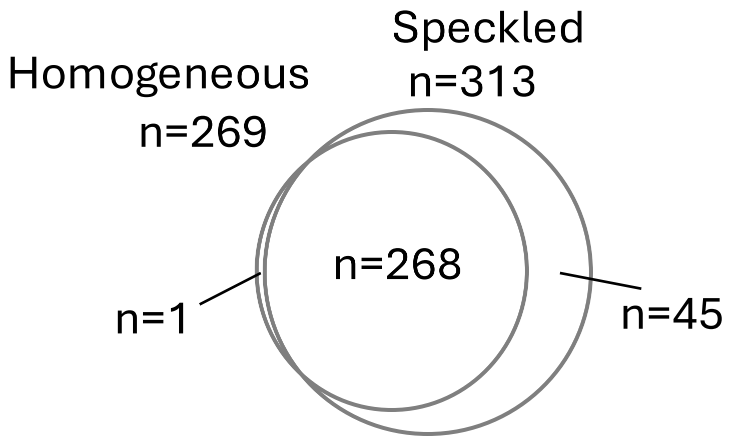

Table S2

Clinical and Laboratory Features by Anti-Nuclear Antibody Staining Patterns in ANA-Positive Patients with Rheumatoid Arthritis

	Speckled			Homogeneous			Nucleolar			Discreate-Speckled		
	Positive	Negative	p-value	Positive	Negative	p-value	Positive	Negative	p-value	Positive	Negative	p-value
Number	313	25		269	69		19	319		16	322	
Gender: Female	247 (78.9)	20 (80.0)	1.00	213 (79.2)	54 (78.3)	0.87	15 (79.0)	252 (79.0)	1.00	15 (93.8)	252 (78.3)	0.21
Age	66 (55, 75)	69 (60, 79)	0.23	66 (55, 75)	70 (60, 77)	0.10	72 (61, 82)	66 (55, 75)	0.03	70 (60, 80)	66 (55, 75)	0.26
Disease duration, M	137 (62, 203)	136 (48, 238)	0.97	140 (61, 203)	133 (57, 213)	0.97	136 (50, 213)	137 (63, 203)	0.89	166 (80, 273)	137 (61, 203)	0.37
BMI	22.5 (20.1, 25.3)	22.9 (20.7, 26.0)	0.63	22.6 (20.1, 25.4)	22.0 (19.8, 25.3)	0.53	22.5 (19.5, 26.0)	22.5 (20.1, 25.3)	0.95	23.4 (20.8, 26.2)	22.5 (20.1, 25.3)	0.44
Family history of RA	66 (21.2)	6 (25.0)	0.61	60 (22.3)	12 (18.2)	0.51	2 (11.1)	70 (22.1)	0.38	4 (25.0)	68 (21.3)	0.76
Smoking history	114 (36.4)	13 (52.0)	0.14	100 (37.2)	27 (39.1)	0.78	8 (42.1)	119 (37.3)	0.81	6 (37.5)	121 (37.6)	1.00
Positivity of RF	259 (82.8)	14 (56.0)	0.003	219 (81.4)	54 (78.3)	0.61	14 (73.7)	259 (81.2)	0.38	9 (56.3)	264 (82.0)	0.02
Positivity of ACPA	258 (82.4)	19 (76.0)	0.42	220 (81.8)	57 (82.6)	1.00	15 (80.0)	262 (82.1)	0.76	12 (75.0)	265 (82.3)	0.50
Erosive X-ray	168 (54.0)	10 (40.0)	0.21	143 (53.4)	35 (51.5)	0.79	8 (42.1)	170(53.6)	0.35	6 (37.5)	172 (53.8)	0.30
Pain VAS	20 (2, 40)	15 (3, 30)	0.64	20 (4, 40)	15 (0, 40)	0.76	10 (0, 30)	20 (2, 40)	0.38	18 (6, 38)	20 (2, 40)	0.68
Fatigue VAS	30 (10, 50)	20 (0, 40)	0.38	30 (10, 50)	20 (3,45)	0.25	10 (0, 40)	30 (10, 50)	0.11	30 (3, 55)	25 (10, 50)	0.96
PGA	20 (10, 40)	20 (0, 30)	0.82	20 (10, 40)	20 (0, 50)	0.72	10 (0, 30)	20 (10, 40)	0.34	30 (3, 45)	20 (10, 40)	0.47
EGA	10 (0, 10)	10 (0, 20)	0.74	10 (0, 10)	10 (0, 10)	0.65	0 (0, 15)	10 (0, 10)	0.73	10 (0, 20)	10 (0, 10)	0.27
TJ	0 (0, 0)	0 (0, 0)	0.44	0 (0, 0)	0 (0, 0)	0.25	0 (0, 0)	0 (0, 0)	0.94	0 (0, 0)	0 (0, 0)	0.36
SJ	0 (0, 1)	0 (0, 0)	0.55	0 (0, 0)	0 (0, 1)	0.03	0 (0, 0)	0 (0, 1)	0.36	0 (0, 0)	0 (0, 1)	0.63
CDAI	3 (1, 7)	3 (0.5, 6)	0.58	3 (1, 7)	3 (1, 6)	0.85	2.5 (0, 6)	3 (1, 7)	0.30	4 (1, 7)	3 (1, 7)	0.61
HAQ	0 (0, 0.63)	0 (0, 1.13)	0.67	0 (0, 056)	0.13 (0, 0.88)	0.26	0.13 (0, 0.88)	0 (0, 0.63)	0.34	0.25 (0, 1.53)	0 (0, 1.38)	0.34
EQ-5D	0.77 (0.65, 1)	1 (0.6, 1)	0.90	0.77 (0.66, 1)	0.77 (0.65, 1)	0.70	0.77 (0.6, 1)	0.77 (0.65, 1)	0.52	0.77 (0.6, 1)	0.77 (0.65, 1)	0.60
D2T-RA	9 (2.9)	1 (4.0)	0.54	7 (2.6)	3 (4.4)	0.43	0 (0)	10 (3.1)	1.00	2 (12.5)	8 (2.5)	0.08
Complication												
Lung disease	37 (11.8)	4 (16.0)	0.52	30 (11.2)	11 (15.9)	0.30	7 (36.8)	34 (10.7)	0.004	1 (6.3)	40 (12.4)	0.70
Malignancy	52 (16.6)	5 (20.0)	0.59	45 (16.7)	12 (17.4)	0.86	5 (26.3)	52 (16.3)	0.34	2 (12.5)	55 (17.1)	1.00
Osteoarthritis	67 (21.4)	6 (24.0)	0.80	58 (21.6)	15 (21.7)	1.00	5 (26.3)	68 (21.3)	0.57	5 (31.3)	68 (21.1)	0.35
Mental disorders	17 (5.4)	0 (0)	0.63	15 (5.6)	2 (2.9)	0.54	1 (5.3)	16 (5.0)	1.00	0 (0)	17(5.3)	1.00
Laboratory data												
RF titer	58 (22, 180)	28 (0, 102)	0.01	56 (21, 180)	51 (21, 141)	0.49	42 (10, 166)	56 (21, 162)	0.63	17 (0, 146)	58 (22, 168)	0.07
ACPA titer	92 (13.0, 468.7)	22.8 (3.2, 173.4)	0.02	79 (11, 419)	109 (8, 433)	0.68	23 (6, 193)	90 (11, 459)	0.10	50 (2, 309)	83 (11, 449)	0.24
ESR	19 (8, 34)	17 (6, 28)	0.33	17 (8, 33)	19 (8, 35)	0.61	18 (7, 34)	18 (8, 33)	0.86	18 (10, 31)	18 (8, 33)	0.85
CRP	0.13 (0.05, 0.34)	0.12 (0.08, 0.27)	0.97	0.12 (0.06, 034)	0.16 (0.06, 0.33)	0.54	0.12 (0.04, 0.23)	0.13 (0.06, 0.34)	0.35	0.12 (0.09, 0.41)	0.12 (0.05, 0.33)	0.48
WBC	5620 (4375, 6905)	5700 (4645, 6755)	0.50	5510 (4308, 6860)	5840 (4785, 7970)	0.18	4990 (4500, 5970)	5655 (4390, 6943)	0.28	6120 (5325, 7088)	5510 (4380, 6900)	0.12
Hb	13.1 (12.1, 13.9)	13.2 (12.1, 14.4)	0.64	13.1 (12.1, 13.9)	13.1 (12, 14.1)	0.79	13.5 (10.9, 14.2)	13.1 (12.1, 13.9)	0.79	13.0 (12.1, 13.4)	13.1 (12.1, 14)	0.63
Plt	231 (190, 278)	229 (189, 257)	0.60	233 (190, 277)	224 (183, 273)	0.30	202 (173, 283)	232 (191, 273)	0.14	226 (193, 249)	232 (189, 279)	0.42
TP	7.2 (7, 7.5)	7.1 (6.9, 7.4)	0.16	7.2 (7, 7.5)	7.1 (6.9, 7.6)	0.25	7.1 (6.9, 7.4)	7.2 (6.9, 7.5)	0.13	7.1 (6.9, 7.6)	7.2 (7, 7.5)	0.66
Alb/Glob ratio	1.3 (1.2, 1.5)	1.4 (1.2, 1.6)	0.25	1.3 (1.2, 1.5)	1.3 (1.1, 1.5)	0.28	1.33 (1.1, 1.5)	1.3 (1.2, 1.5)	0.82	1.3 (1.1, 1.5)	1.3 (1.2, 1.5)	0.90
IgG	1361 (1149, 1603)	1270 (1007, 1511)	0.11	1341 (1141, 1592)	1405 (1111, 1645)	0.53	1309 (1097, 1479)	1356 (1145, 1613)	0.33	1326 (921, 1638)	1353 (1148, 1598)	0.56
IgA	279 (205, 359)	245 (195, 301)	0.23	270 (207, 352)	259 (190, 368)	0.79	268 (218, 363)	266 (204, 354)	0.75	244 (190, 299)	269 (205, 359)	0.21
IgM	104 (76, 145)	72 (58, 122)	0.03	103 (77, 141)	96 (57, 145)	0.15	72 (54, 112)	104 (76, 146)	0.01	98 (65, 153)	102 (75, 141)	0.64
Previously Used DMARDs												
MTX	270 (86.3)	24 (96.0)	0.22	235 (87.4)	59 (85.5)	0.69	15 (79.0)	279 (87.5)	0.29	15 (93.8)	279 (86.7)	0.70
bDMARDs	196 (62.6)	15 (60.0)	0.83	166 (61.7)	45 (65.2)	0.68	13 (68.4)	198 (62.1)	0.63	10 (62.5)	201 (62.4)	1.00
TNFi	168 (54.0)	11 (44.0)	0.41	143 (53.2)	36 (52.2)	0.89	9 (47.4)	170 (53.3)	0.64	7 (43.8)	172 (53.4)	0.61
IL-6Ri	56 (17.9)	3 (12.0)	0.59	45 (16.7)	14 (20.3)	0.48	5 (26.3)	54 (16.9)	0.35	1 (6.3)	58 (18.0)	0.32
ABT	42 (13.4)	3 (12.0)	1.00	34 (12.6)	11 (15.9)	0.55	5 (26.3)	40 (12.5)	0.15	4 (25.0)	41 (12.7)	0.25
JAKi	47 (15.0)	4 (16.0)	0.78	43 (16.0)	8 (11.6)	0.45	6 (31.6)	45 (14.1)	0.05	0 (0)	51 (15.8)	0.14
GC	195 (62.3)	17 (68.0)	0.67	167 (62.1)	45 (65.2)	0.68	11 (57.9)	201 (63.0)	0.64	9 (56.3)	203 (63.0)	0.60
DMARDs in Current Use												
MTX	188 (60.1)	15 (60.0)	1.00	162 (60.2)	41 (59.4)	1.00	11 (57.9)	192 (60.2)	1.00	9 (56.3)	194 (60.3)	0.80
TNFi	82 (26.2)	4 (16.0)	0.34	73 (27.1)	13 (18.8)	0.17	1 (5.3)	85 (26.6)	0.05	3 (18.8)	83 (25.8)	0.77
IL-6Ri	29 (9.3)	2 (8.0)	1.00	22 (8.2)	9 (13.0)	0.24	3 (15.8)	28 (8.8)	0.40	1 (6.3)	30 (9.3)	1.00
ABT	23 (7.4)	2 (8.0)	0.71	18 (6.7)	7 (10.1)	0.31	2 (10.5)	23 (7.2)	0.64	2 (12.5)	23 (7.1)	0.33
JAKi	39 (12.5)	4 (16.0)	0.54	35 (13.0)	8 (11.6)	0.84	6 (31.6)	37 (11.6)	0.02	0 (0)	43 (13.4)	0.24
GC	53 (16.9)	2 (8.0)	0.40	44 (16.4)	11 (15.9)	1.00	2 (10.5)	53 (16.6)	0.75	2 (12.5)	53 (16.5)	1.00
